# Association study of lipoprotein(a) genetic markers, traditional risk factors, and coronary heart disease in HIV-1-infected patients

**DOI:** 10.3389/fimmu.2012.00367

**Published:** 2012-12-06

**Authors:** Lander Egaña-Gorroño, Esteban Martínez, Tuixent Escribà, Marta Calvo, José M. Gatell, Mireia Arnedo

**Affiliations:** ^1^Group of Genomics and Pharmacogenomics in HIV, Retrovirology and Viral Immunopathology Laboratory, Institut D’Investigacions Biomèdiques August Pi i Sunyer, Hospital Clínic BarcelonaBarcelona, Spain; ^2^Department of Infectious Diseases, Hospital Clínic, Faculty of Medicine, University of BarcelonaBarcelona, Spain

**Keywords:** HIV, coronary heart disease, host genetics, copy number variation, *LPA* gene

## Abstract

**Objectives:** General population studies have shown associations between copy number variation (CNV) of the *LPA* gene Kringle-IV type-2 (KIV-2) coding region, single-nucleotide polymorphism (SNP) rs6415084 in *LPA* and coronary heart disease (CHD). Because risk factors for HIV-infected patients may differ from the general population, we aimed to assess whether these potential associations also occur in HIV-infected patients. **Methods:** A unicenter, retrospective, case–control (1:3) study. Eighteen HIV-patients with confirmed diagnosis of acute myocardial infarction (AMI) were adjusted for age, gender, and time since HIV diagnosis to 54 HIV-patients without CHD. After gDNA extraction from frozen blood, both CNV and SNP genotyping were performed using real-time quantitative PCR. All genetic and non-genetic variables for AMI were assessed in a logistic regression analysis. **Results:** Our results did not confirm any association in terms of lipoprotein(a) *LPA* structural genetic variants when comparing KIV-2 CNV (*p* = 0.67) and SNP genotypes (*p* = 0.44) between AMI cases and controls. However, traditional risk factors such as diabetes mellitus, hypertension, and CD4^+^ T cell count showed association (*p* < 0.05) with CHD. **Conclusion:** Although significant associations of AMI with diabetes, hypertension and CD4^+^ T cell count in HIV-patients were found, this study could not confirm the feasibility neither of KIV-2 CNV nor rs6415084 in *LPA* as genetic markers of CHD in HIV-infected patients.

**Highlights**:

● Individuals with HIV infection are at higher risk of coronary heart disease (CHD) than the non-infected population.

● Our results showed no evidence of *LPA* structural genetic variants associated with CHD in HIV-1-infected patients.

● Associations were found between diabetes mellitus, arterial hypertension, CD4^+^ T cell count, and CHD.

● The clinical usefulness of these biomarkers to predict CHD in HIV-1-infected population remains unproven.

● Further studies are needed to assess the contribution of common genetic variations to CHD in HIV-infected individuals.

## INTRODUCTION

Since the first case reports of acute myocardial infarction (AMI) in HIV-1-infected patients on highly active antiretroviral therapy (HAART) were described, it has become increasingly evident that individuals with HIV infection are at higher risk of cardiovascular events than the general population (Triant et al., 2007). Besides, the contribution of classic risk factors, other factors such as viral replication, HIV-associated inflammation and/or immunodeficiency, and antiretroviral therapy have been associated with premature cardiovascular disease (CVD; Friis-Møller et al , 2003; Hansson, 2005). The study of genetic traits and their relation to complex diseases, e.g., CVD, still poses a major challenge.

Increased plasma concentrations of atherogenic lipoproteins play an important role in the development of atherosclerosis leading to premature AMI and ischemic stroke. One particular fraction, which is important in that respect, is lipoprotein(a) [Lp(a)]. Elevated plasma concentrations of Lp(a) have been associated with the risk of coronary heart disease (CHD) in the general population (Rhoads et al., 1986; Ridker et al., 1993; Ohira et al., 2006). The plasma concentration of Lp(a) varies over a wide range among individuals. Furthermore, the inter-individual variation in Lp(a) level is 90% genetically determined by the *LPA* locus on chromosome 6, although plasma Lp(a) in a particular individual remains stable over a lifetime (Boerwinkle et al., 1992).

Lipoprotein(a) is composed of an apolipoprotein(a) [Apo(a)] molecule encoded by *LPA*, which is connected via a disulphide bond to the apolipoprotein B-100 of a proatherogenic LDL-cholesterol particle (McLean et al., 1987; Berglund and Ramakrishnan, 2004). The Apo(a) molecule is composed of a signal peptide region, 10 types of kringles that differ in sequence but are homologous to plasminogen kringle IV (KIV1–10), a kringle homologous to plasminogen kringle V (KV) and an inactive protease-like domain (McLean et al., 1987; Gavish et al., 1989). The size of Apo(a) is determined by a copy number variation (CNV) of the kringle-IV type 2 (KIV-2) coding region (encoded by exons 4 and 5 in *LPA* gene), and this being negatively correlated with Lp(a) levels (Lanktree et al., 2009). The genetically determined KIV-2 repeat number affects the final size of the Apo(a) protein, with larger isoforms being compromised with respect to protein folding, transport, and secretion (Lanktree et al., 2010). Furthermore, the *LPA* single-nucleotide polymorphism (SNP) rs6415084 (C → T), within the same haplotype block as the KIV-2 CNV, has been reported to be significantly associated with both Lp(a) concentrations and the KIV-2 copy number (CN; Paultre et al., 2000; Clarke et al., 2009). Several studies have identified increased atherogenesis and CHD in individuals with fewer apo(a) KIV-2 repeats (Kraft et al., 1992, 1996; Sandholzer et al., 1992). The number of KIV-2 repeats varies among subjects and ranges from 11 to >50 (Kannel et al., 1976; Lackner et al., 1993). Contrary to KIV-2 with CN > 25, a CN ≥22 leads to significantly higher Lp(a) levels, which are more frequent in CHD patients (Sandholzer et al., 1992; Kraft et al., 1996). The aim of this study was to test whatever association between KIV-2 CNV and CHD could be confirmed in HIV-infected patients, as well as association between classical clinical risk factors and CHD.

## MATERIALS AND METHODS

The study population consisted of 72 HIV-1-infected individuals under HAART who were followed-up at the Hospital Clínic of Barcelona during the study period (January 1, 1997 to December 31, 2008). They had all signed the ethical informed consent for genetic testing. The retrospective case–control study (1:3) was based on CHD patients from whom genetic testing could be performed (*n* = 18). CHD was defined according to the criteria of the Joint European Society of Cardiology and the American College of Cardiology Committee for the Redefinition of Myocardial Infarction (The Joint European Society of Cardiology/American College of Cardiology Committee, 2000). Patients with CHD, had either an ST-segment elevation myocardial infarction (*n* = 11, 61%) or a non-ST-segment elevation myocardial infarction (*n* = 7, 39%) if there was or not an evolving ST-segment elevation >0.1 mV in two contiguous leads, respectively. Each case was matched by age, gender, and time since HIV diagnosis to three controls without CHD (*n* = 54). Besides age and gender, relevant data on HIV infection and on clinical risk factors for CVD were collected. DNA was isolated from frozen blood according to manufacturer’s instructions using the QIAamp DNA Blood Mini Kit, automated by QIAcube (QIAGEN). For the CNV analysis a multiplex qPCR was carried out using custom TaqMan probes for exons 4 and 5 in *LPA* and single-copy reference gene *RNaseP* (part number 4316844) in the Applied Biosystems 7900HT Fast Real-time PCR System. Reaction volumes contained 4 µl of water, 1 µl of 20× TaqMan primer/probe mix for *LPA*, 1 µl of 20× TaqMan primer/probe mix for *RNaseP*, 10 µl of 2× Genotyping Master Mix (Applied Biosystems), and 4 µl of genomic DNA at a final concentration of 5–10 ng. Six replicates were run in all qPCRs. Thermocycler conditions were as follows: 95°C hot-start for 10 min, followed by 40 cycles at 95°C for 15 s and 60°C for 1 min. Absolute quantification (AQ) files were then exported to the Copy Caller Software (Applied Biosystems) for relative quantifications (RQ) and final CN analysis.

Intronic SNP rs6415084 in *LPA* gene was genotyped by TaqMan Allelic Discrimination, using TaqMan SNP genotyping assays predesigned by Applied Biosystems (part number C_27422575_10). Reaction volumes contained 1.25 µl of 20× TaqMan SNP Assay, 12.5 µl of 2× Universal Master Mix (Applied Biosystems), and 11.25 µl of genomic DNA at a final concentration of 1–20 ng. Thermocycler conditions were as follows: 95°C hot-start for 10 min, followed by 40 cycles at 95°C for 15 s and 60°C for 1 min. 15% of the total samples were re-genotyped in order to check reproducibility.

A basic case/control association test, based on a Fisher’s exact test, was performed by comparison of calculated CN variants and SNP genotypes between AMI cases and controls using SNPator software (CEGEN, Barcelona, Spain). A multivariate analysis through logistic regression model was performed in order to assess the influence of genetic and non-genetic variables using SPSS v.15. With the sample size available, any difference above 20% would have been detected with a power of 80%.

## RESULTS

Baseline characteristics of the study participants are shown in **Table 1**. The median age was 44 years (interquartile range 12). Men represented 88.9% in both groups. Groups of risk for HIV acquisition were 50% homosexuals, 25% heterosexuals, and 25% injection drugs users in both groups. The median CD4^+^ T cell count (cell/µl) was 107 (interquartile range 261) in the cases and 210 (interquartile range 275) in the controls. HIV viral load (log) was 4.81 (interquartile range 5.92) in the cases and 4.62 (interquartile range 5.34) in the controls. A total of 88.9% of cases and 64.8% of controls were exposed to protease inhibitor containing regimens. Diabetes mellitus and usual smokers were present in 22.2 and 55.5% of cases and 14.8 and 37.03% of controls, respectively. A total of 22.2% of cases and 9.25% of controls had hypertension, and 22.2% of cases and 3.7% of controls had family antecedents of CVD. At least, one AIDS event was suffered by 2 (11.1%) cases and 12 (22.2%) controls. Both groups had a median Framingham risk score (FRS) of 2% (interquartile range 14 in cases and 5.5 in controls).

**Table 1:** Baseline characteristics of the study participants.

Characteristic	Study participants (*n* = 72)	
	AMI (*n* = 18)	No AMI (*n* = 54)
Age, years*	44 (12)	42 (12)
Men/women, *n* (% men)	16 (88.9)	48 (88.9)
**Ethnicity, *n* (%)**		
Caucasian	16 (88.9)	52 (96.3)
Unknown/other	2 (11.1)	2 (3.7)
**Presumed mode of HIV transmission** ***n*** **(%)**		
Men who have sex with men	9 (50)	26 (48.1)
Heterosexual	4 (22.2)	14 (25.9)
Injection drug user	5 (27.8)	10 (18.5)
Other/unknown	0 (0)	4 (7.4)
**Follow-up period**		
***At HIV diagnostic time:***		
CD4^+^ T cell count (cell/µl)*	107 (261)	210 (275)
HIV Viral load (log)*	4.81 (5.92)	4.62 (5.34)
***At time of event:***		
CD4^+^ T cell count (cell/µ)*	573.5 (377)	-
HIV Viral load (log)*	2.99 (3.96)	-
Exposure to PI, *n* (%)	16 (88.9)	35 (64.8)
Exposure to other HAART regimens, *n* (%)	2 (11.1)	19 (35.2)
Smokers, *n* (%)	10 (55.5)	20 (37.03)
Diabetes mellitus, *n* (%)	4 (22.2)	8 (14.8)
Hypertension, *n* (%)	4 (22.2)	5 (9.25)
Familial antecedents, *n* (%)	4 (22.2)	2 (3.7)
AIDS events, *n* (%)	2(11.1)	12 (22.2)
Framingham risk score (%)*	2 (14)	2 (5.5)

*AMI, acute myocardial infarction; PI, protease inhibitor; HAART, highly active antiretroviral therapy.*

* Median (interquartile range).

All participants were successfully genotyped. As it was expected, the *LPA* KIV-2 repeat analysis ranged between 17 and 48 copies. The case/control analysis showed CN ≥22 in seven patients (one case vs. six controls) and CN > 25 in 65 patients [17 cases vs. 48 controls; *p* = 0.46; OR = 0.44 (0.049–3.99)]. SNP rs6415084 was checked to confirm Hardy–Weinberg equilibrium. Genotyping assay showed common homozygosity (C/C) in 17 patients (3 cases vs. 14 controls), rare homozygosity (T/T) in 20 patients (7 cases vs. 13 controls), and heterozygosity (C/T) in 35 patients [8 cases vs. 27 controls; *p* = 0.42; OR = 0.57 (0.144, 2.274)].

Logistic regression analysis showed significant associations (*p* < 0.05) between traditional risk factors such as diabetes mellitus, hypertension, and CD4^+^ T cell count at HIV-infected diagnosis and CHD (**Table 2**). The strength of the association was quantified using the odds ratios and the 95% confidence intervals.

**Table 2:** Multivariate analysis through logistic regression including all genetic and non-genetic co-variables.

Co-variable	*p*-Value	OR	95% CI	
			Lower	Upper
PI	0.906	8.68	0.46	12.87
Smoker	0.277	2.044	0.563	7.424
Diabetes mellitus	0.024*	2.44	2.3	5.78
Hypertension	0.035*	3.576	2.52	3.96
Familial antecedents	0.74	6.912	0.829	5.764
AIDS events	0.569	0.603	0.105	3.449
Viral load (log)	0.368	1.204	0.152	1.965
CD4^+^ T cell count (cell/µ)	0.042*	1.22	1.06	1.047
**Genetic variables:**
rs6415084	0.528	0.594	0.118	2.987
KIV-2 CNV	0.776	1.015	0.914	1.128

*PI, protease inhibitor; CNV, copy number variation; OR, odd ratio; 95% CI, 95% confidence interval;*

* p < 0.05.

## CONCLUSION

The most important finding in our study is that we were able to detect associations between clinical risk factors and CHD. Non-genetic variables such us diabetes mellitus, hypertension, and CD4^+^ T cell count at HIV-infected diagnosis reached significance in the logistic regression analysis. The influence of CD4^+^ T cell level on CVD is supported by previously reported studies (Paultre et al., 2000). However, any previously described associations between *LPA* genetics biomarkers (KIV-2 CNV nor rs6415084) and CHD were found. The association between SNPs in *LPA* gene and circulating Lp(a) levels, and consequently with CHD, may be mediated by various mechanisms: (i) some of the SNPs may be in linkage disequilibrium with the KIV-2 repeat polymorphism which has been shown to explain approximately 50% of the genetic variation in Lp(a) concentrations; (ii) certain SNPs may directly influence the transcriptional and/or translational processes of the *LPA* gene (Paultre et al., 2000); and (iii) some non-causal SNPs may be in linkage disequilibrium with SNPs having causal effect on Lp(a) concentrations. The main important limitation of our study was the low absolute number of HIV-patients with documented AMI. Furthermore, larger number of individuals could not be included in the study because of the unavailability of data or blood sample for the purpose of this study. Lp(a) concentrations from frozen blood could not be measured. Although the power of the study was limited due to the small sample size analyzed, there is no other study regarding CNV in *LPA* and CHD in HIV-infected patients.

In summary, our results did not confirm evidence of *LPA* structural genetic variants associated with CHD in HIV-1-infected patients. Although the power of the study was limited, traditional risk factors are contributing to the progression of CHD amongst HIV-infected population as well as they do in non-HIV population. Moreover, there are factors in HIV-infected population that are not present in non-HIV-infected individuals and are unrelated to genetics: HIV and HAART. The pathogenesis of HIV-1-infection and/or exposure to HAART might contribute to CHD in a stronger way than *LPA* genetic variants do. The clinical utility of these biomarkers to predict CHD in HIV-1-infected population therefore remains unproven. Further collaborative studies with a larger number of HIV-infected patients are needed.

## Conflict of Interest Statement

The authors declare that the research was conducted in the absence of any commercial or financial relationships that could be construed as a potential conflict of interest.
